# Students’ Achievement Goals, Learning-Related Emotions and Academic Achievement

**DOI:** 10.3389/fpsyg.2016.00603

**Published:** 2016-05-02

**Authors:** Marko Lüftenegger, Julia Klug, Katharina Harrer, Marie Langer, Christiane Spiel, Barbara Schober

**Affiliations:** Department of Applied Psychology: Work, Education and Economy, University of ViennaVienna, Austria

**Keywords:** achievement goal, emotion, boredom, achievement, enjoyment

## Abstract

In the present research, the recently proposed 3 × 2 model of achievement goals is tested and associations with achievement emotions and their joint influence on academic achievement are investigated. The study was conducted with 388 students using the 3 × 2 Achievement Goal Questionnaire including the six proposed goal constructs (task-approach, task-avoidance, self-approach, self-avoidance, other-approach, other-avoidance) and the enjoyment and boredom scales from the Achievement Emotion Questionnaire. Exam grades were used as an indicator of academic achievement. Findings from CFAs provided strong support for the proposed structure of the 3 × 2 achievement goal model. Self-based goals, other-based goals and task-approach goals predicted enjoyment. Task-approach goals negatively predicted boredom. Task-approach and other-approach predicted achievement. The indirect effects of achievement goals through emotion variables on achievement were assessed using bias-corrected bootstrapping. No mediation effects were found. Implications for educational practice are discussed.

## Introduction

Achievement goals and emotions are crucial determinants of students’ learning processes and have an impact on academic outcomes (Hulleman et al., 2010; Goetz and Hall, 2013). The interplay between achievement goals and affect in academic settings has been a part of achievement goal theory since its beginnings (Dweck and Leggett, 1988). However, empirical educational research on the relation between achievement goals and discrete emotions was sparse for a long time. Although the last decade has brought a discernible increase in empirical research on the specific relationship between goals and discrete emotions, recent developments in achievement goal theory have to our knowledge not been considered so far. Therefore, the present research tests the recently proposed 3 × 2 model of achievement goals (Elliot et al., 2011) and investigates its associations with activity emotions and academic achievement in a comprehensive model.

### Achievement Goals

Research on achievement goals has a long tradition in educational research and has resulted in the development of various conceptual models. The dichotomous model (Nicholls, 1984; Dweck, 1986; Maehr, 1989; Ames, 1992) distinguishes between two goal types: mastery goals (developing competence) and performance goals (demonstrating competence). These two types of goals are also referred to as learning goals and performance goals (Dweck and Leggett, 1988), task or ability goals (Maehr, 1989), or task-involvement goals and ego-involvement goals (Nicholls, 1984). Although there has been some disagreement as to whether these pairs all represent similar constructs (Thorkildsen and Nicholls, 1998), most researchers today see enough overlap to treat them in a similar fashion (Schunk et al., 2008). The trichotomous model (Elliot and Harackiewicz, 1996) and the 2 × 2 model (Elliot, 1999) incorporate the approach-avoidance distinction into the two goal types. Furthermore, developments in achievement goal research have led to a change in how the striving for competence is defined. Nowadays, a goal is defined as “a future-focused cognitive representation that guides behavior to a competence-related end state that the individual is committed to either approach or avoid” (Hulleman et al., 2010, p. 423). In the newly developed 3 × 2 conceptualization, achievement goals are differentiated along the definition component (task-based, self-based, and other-based) and the valence component (approaching success, avoiding failure) of competence. This conceptualization encompasses six goal constructs (task-approach, task-avoidance, self-approach, self-avoidance, other-approach, and other-avoidance). Research on the 3 × 2 achievement goal framework has been scarce so far, with a particular dearth of research focused on students. In addition to the original study of Elliot et al. (2011), empirical proof of the factorial validity of the 3 × 2 model for students was reported in a Norwegian undergraduate sample (Diseth, 2015). Four studies (Johnson and Kestler, 2013; Diseth, 2015; Gillet et al., 2015; Stoeber et al., 2015) have investigated the predictive validity of the 3 × 2 model in testing associations with relevant learning outcomes such as perfectionism, self-efficacy, value, learning strategies, and academic achievement. The 3 × 2 framework has also been recently applied to the sports domain (Mascret et al., 2015a) and to teachers (Mascret et al., 2015b). Reproducibility is a core principle of scientific progress (Asendorpf et al., 2013), especially for new scientific claims/theories. However, recent findings have shown that replication rates in psychological research are below 40% (Open Science Collaboration, 2015), and even in cases where replication is successful, replication effects averaged just half the magnitude of the original effects. Against this background, the first aim of the present study was to replicate the factor structure of the 3 × 2 model including six goal types (task-approach, task-avoidance, self-approach, self-avoidance, other-approach, and other-avoidance) to provide more empirical evidence for the newest achievement goal conceptualization.

### Achievement Emotions

In relation to achievement goals, achievement emotions are of special interest (Pekrun, 2006). Achievement emotions reflect the temporary affective state of a learner when performing a learning-related task in a school, college or university setting. Learning-related emotions appear in different academic settings, such as attending class, studying and taking exams. Emotions can vary across these settings. Using a three-dimensional taxonomy (Pekrun, 2006), learning related emotions can be differentiated by valence, object focus, and activation. In terms of valence, positive emotions (e.g., enjoyment) are distinguished from negative emotions (e.g., anxiety, boredom). Object focus concerns whether the emotion is experienced in relation to a study activity itself or to the achievement of an academic goal (=outcome). Outcome-focused emotions can be prospective (e.g., hope) or retrospective (e.g., pride). The third dimension, activation, refers to the degree of physiological arousal involved in that emotion (e.g., hope is activating and hopelessness is deactivating). In the present study, we focus on the activity emotions of enjoyment (positive, activating) and boredom (negative, deactivating) while learning for an exam. The reasons are threefold. First, both of these emotions are frequently experienced by students in achievement settings (Pekrun et al., 2002). Second, they differ systematically in terms of valence (positive vs. negative) and activation (activating vs. deactivating), both of which are assumed to be pivotal to examining the effects of emotions on achievement. Third, empirical evidence suggests that enjoyment and boredom are related to both mastery and performance goals (for an overview see Linnenbrink-Garcia and Barger, 2014).

### Achievement Goals and Emotions

In theoretical conceptualizations, discrete emotions are seen as outcomes of goal pursuit, whereas general affect, temperament, or moods are instead seen as antecedents of goal adoption (e.g., Seifert, 1995; Elliot and Thrash, 2002; Linnenbrink and Pintrich, 2002; Pekrun et al., 2006, 2009). There are already a number of empirical studies on the relationship between goals and emotions that use a trichotomous (e.g., Pekrun et al., 2006, 2009; Daniels et al., 2009) or 2 × 2 goal conceptualization (e.g., Putwain et al., 2013). These results show that achievement goals and emotions are intricately related (for an overview see Linnenbrink-Garcia and Barger, 2014). Meta-analytic evidence (Huang, 2011) shows significant mean correlations between achievement emotions and mastery approach goals (0.20), mastery avoidance goals (-0.24) and performance avoidance goals (-0.20). Moreover, findings from Huang’s meta-analysis (2011) indicate a general pattern of positive academic emotions (such as enjoyment, hope and interest) corresponding to mastery-approach (mean correlation of 0.42) and performance-approach goals (0.14), and negative emotions (such as anxiety, anger, and sadness) corresponding to mastery-avoidance (-0.38) and performance-avoidance goals (-0.31).

Focusing on our study variables, mastery-approach goals are consistently positively related to enjoyment and negatively related to boredom (Pekrun et al., 2006, 2009; Daniels et al., 2008, 2009; King et al., 2012; Goetz et al., 2016). For mastery-avoidance goals, only sparse empirical findings are available and there is a clear need for additional research. The picture for performance-approach goals is more complex. They are in some studies related to enjoyment (Daniels et al., 2008; King et al., 2012). Negative emotions relate less consistently to performance-approach goals: studies have found positive, negative and no relation at all. Performance-avoidance goals are positively related to negative deactivated emotions such as boredom (Pekrun et al., 2009, Study 2) and consistently unrelated to enjoyment (Pekrun et al., 2006, 2009). To our knowledge, no other study has tested the relationship of the six 3 × 2 model goal constructs with enjoyment and boredom.

### Goals and Emotions Predicting Academic Achievement

#### Goals and Achievement

Performance-avoidance goals are quite consistently negatively associated with achievement (e.g., Elliot, 2005), whereas performance-approach goals are often positively linked to achievement (Martin, 2013). Concerning mastery-approach goals, the evidence is mixed, with some research finding no significant association with achievement (see Anderman and Wolters, 2006 for a summary) and other research suggesting a significant positive connection under particular conditions such as experimental settings (Linnenbrink-Garcia et al., 2008) or for items containing no goal-relevant language (Hulleman et al., 2010). Research using the 3 × 2 model with college students showed that achievement was positively related to other-approach goals (Elliot et al., 2011; Diseth, 2015) and negatively related to other-avoidance goals (Elliot et al., 2011; Johnson and Kestler, 2013) and self-approach goals (Diseth, 2015).

#### Emotions and Achievement

Achievement emotions can affect students’ achievement (for an overview see Goetz and Hall, 2013). Pleasant activating emotions, such as enjoyment, are expected to positively affect academic achievement. Conversely, unpleasant deactivating emotions, such as boredom, are claimed to decrease motivation and the elaborate processing of information, thus implying negative effects on academic achievement (Pekrun, 2006). These proposed effects were reported in empirical studies with undergraduate students (Daniels et al., 2009; Pekrun et al., 2009, 2014b).

#### Emotions as Mediators of the Relation between Goals and Achievement

Within an achievement goal framework, goals are conceptualized as having a direct effect on psychological processes relevant to achievement, thereby influencing it. Emotions are considered to be one such psychological process mediating the effect of achievement goals on achievement (Elliot and McGregor, 1999; Linnenbrink et al., 1999; Pekrun et al., 2006, 2009). Only a few studies have focused on the mediational role of emotions for goal effects on academic achievement (e.g., Daniels et al., 2009; Pekrun et al., 2009) and clear empirical evidence, especially considering the 3 × 2 model, does not yet exist. In testing the mediational effect, we use advanced statistical methods, including a structural equation framework and a rigorous bootstrap test for significance testing, to overcome the reported flaws (Zhao et al., 2010) of the classical mediation approach (Baron and Kenny, 1986) used in former studies.

### Aims of the Present Study

The 3 × 2 achievement goal model is one of the newest conceptualizations in goal theory, meaning that clear empirical evidence of its replicability does not yet exist. Additionally, there are a lack of empirical studies examining the associations between the new six goal types and related variables in prior work on goals, such as achievement emotions and academic achievement. Therefore, the aims of the present research were: first, to replicate the findings of Elliot et al. (2011) regarding the factor structure of the 3 × 2 model including six goal types (task-approach, task-avoidance, self-approach, self-avoidance, other-approach, and other-avoidance); second, to examine the associations between the six goal types and activity emotions (enjoyment, boredom); and third, to investigate the joint influence of achievement goals and emotions on academic achievement (goals– emotion–achievement linkage).

## Materials and Methods

### Participants and Procedure

The survey was conducted with 388 (82% female) psychology students with a mean age of 25 years (*SD* = 5.1). All students attended the lecture “Research methods and evaluation.” This lecture was obligatory for bachelor (29.8%) and diploma students (70.2%) and sought to teach basic principles of scientific work. Data was collected using an online questionnaire.

The present study was conducted in compliance with ethical standards adopted by the Austrian Federal Ministry of Health (Bundesministerium für Gesundheit, 1995) and the American Psychological Association (American Psychological Association [APA], 2010). Accordingly, prior to participation, students were informed about the goals of the research, duration, procedure and anonymity of their data. Participation in the study was voluntary, informed consent was assured and the students did not receive compensation for their participation in the study. Participants were assured that all of their responses would remain confidential and would not influence their course grade.

### Measures

#### Achievement Goals

Goals were assessed using the German version of the 3 × 2 Achievement Goal Questionnaire (AGQ; Elliot et al., 2011), which included six scales consisting of three items for each goal type (task-approach, task-avoidance, self-approach, self-avoidance, other-approach, and other-avoidance). All items used a 7-point scale, ranging from 1 (strongly disagree) to 7 (strongly agree) and referred to the exam for the “Research methods and evaluation” lecture course.

#### Achievement Emotions

Students’ emotions related to learning for the exam were assessed using short versions of the boredom and enjoyment learning emotions scales from the Achievement Emotions Questionnaire (AEQ; Pekrun et al., 2011). The instructions for the scales assessing the activity emotions enjoyment (eight items) and boredom (eight items) asked respondents to describe how they felt before, during, and after learning for the exam. Participants responded on a scale ranging from 1 (strongly disagree) to 5 (strongly agree), and scores were averaged to form the achievement emotion indexes. The AEQ was validated in several studies and is widely used in the context of education (see Pekrun et al., 2011). Empirical studies indicate that emotion-achievement relations are, on average, weak to moderate in magnitude (Goetz and Hall, 2013). A recent meta-analysis (Tze et al., 2016) revealed a significant and modest negative overall relationship of *r* = −0.24 between boredom and academic outcomes (20 of the 29 studies included in the meta-analysis used the AEQ).

In this study, construct validity of both scales was tested using confirmatory factor analysis (CFA). Fit indices indicated a good model fit for both the enjoyment [χ^2^ (20, *N* = 388) = 49.46; CFI = 0.974; TLI = 0.964; RMSEA = 0.057; SRMR = 0.027] and boredom scales [χ^2^ (20, *N* = 388) = 97.62; CFI = 0.950; TLI = 0.930; RMSEA = 0.100; SRMR = 0.027]. To test for external validity, we analyzed external linkages of both enjoyment and boredom to academic interest (Krapp, 2002). Based on theoretical considerations and empirical findings we expected interest and enjoyment to be positively related (for an overview see Ainley and Hidi, 2014), and interest and boredom to be negatively related (see e.g., Pekrun et al., 2002). Academic interest was assessed using three items from Lüftenegger et al. (2012) and three items from Schiefele et al. (1988). In item formulation different aspects of interest (intrinsic motives, value, emotional valence; Krapp, 2002) were considered (*k* = 6; sample item: “In this course I am learning something that I find to be important”; α = 0.90; composite reliability = 0.91). In our study, interest was positively related to enjoyment (*r* = 0.77) and negatively related to boredom (*r* = −0.49). These results are in line with both theoretical considerations and previous empirical findings thus indicating external validity of the emotion scales.

#### Exam Performance

Students’ final exam grades were used as an indicator of academic achievement. At the end of the semester, an exam was administered consisting of 25 multiple-choice questions assessing both declarative and procedural knowledge. To pass the exam, 51% correct answers were needed. In Austria, grades range from 1 (excellent) to 5 (insufficient/fail), meaning the lower the numerical value, the higher the achievement. For a more comprehensible interpretation of the results, the values were recoded, in a way that 1 represents “insufficient/fail” and 5 represents “excellent.” Therefore, in this study, the higher the numerical value of the grade, the higher the academic achievement.

### Data Preparation and Analytical Approach

All analyses were conducted using the software Mplus 7.31 (Muthén and Muthén, Muthén and Muthén). The rate of individuals omitting items (non-response) was <1% for all considered items and, as such, very low. We used the full information maximum likelihood (FIML) approach implemented in Mplus to deal with missing values. This approach takes all available information into account when estimating the model parameters (Schafer and Graham, 2002).

In a first step, a confirmatory factor analysis (CFA) was conducted to examine the construct validity of the 3 × 2 achievement goal model. In a second step, following previous studies (Elliot et al., 2011; Diseth, 2015; Mascret et al., 2015a,b), we compared the fit of the 3 × 2 model to five alternative achievement goal conceptualizations: (1) a model in which all items loaded onto one latent factor, testing whether a strictly unidimensional conceptualization of achievement goals exists; (2) a dichotomous model in which task-based and self-based goals load together on a joint latent factor and other-based goals load together on another joint latent factor; (3) a trichotomous model in which task-based and self-based goals load together on a joint latent factor, while other-approach and other-avoidance goals load on their hypothesized latent factors; (4) a 2 × 2 model in which approach task-based and self-based goals load together on a joint latent factor, as do avoidance task-based and self-based goals, while other-based goals load on their hypothesized latent factors in this fourth variant; (5) a definition model, in which all items sharing a competence definition load together on joint latent factors (self-based, task-based, and other-based).

Third, multiple mediator modeling (Preacher and Hayes, 2008) with latent variables (structural equation modeling; Kline, 2011) was employed to investigate the associations between goals, emotions, and academic achievement. Two multiple mediator models were compared: (1) without direct associations between goals and achievement, and (2) with direct associations between goals and achievement.

Finally, as part of the structural analyses we also assessed the proposed indirect effects of achievement goals through emotion variables on achievement. The significance of these indirect effects was determined using a bias-corrected bootstrap method with 95% confidence intervals (MacKinnon et al., 2004). This method is superior to the traditional Sobel test, which has been found to be overly conservative and lacking in power even with large samples (MacKinnon et al., 2002). The indirect effect is considered significant if the CI does not include zero. Because we tested direct and indirect effects simultaneously, these calculations can reveal significant indirect effects without a significant direct effect of the predictor variable (e.g., other-approach goal) on the criterion variable (e.g., academic achievement). The presence of mediation is indicated when the indirect effect is significant and the direct effect of the predictor on the criterion variables is non-significant.

Model parameters were estimated via maximum likelihood robust estimation (MLR). MLR is a precise parameter estimator for models that contain continuous non-normal distributed variables. All models were evaluated using fit indices. Following Kline’s (2011) recommendation, we used root mean square error of approximation (RMSEA), Standardized root mean square residual (SRMR), the comparative fit index (CFI), and the Tucker-Lewis index (TLI) in addition to the χ^2^ Test of Model Fit value and its associated degrees of freedom. Although there is little consensus on cutoff values for adequate fit (Lance et al., 2006), we used traditional cutoff scores indicative of excellent and adequate fit to the data, respectively: (a) CFI and TLI ≥ 0.95 and ≥ 0.90, and (b) RMSEA and SRMR ≤ 0.06 and ≤ 0.08. For model comparison, we used the Bayesian information criterion (BIC; Schwarz, 1978), the sample-sized adjusted Bayesian information criterion (SABIC), and the Akaike Information Criterion (AIC), all of which allow for the comparison of competing non-nested models (Burnham and Anderson, 2004).

## Results

Differences with regard to gender and course of study (bachelor, master) were investigated in preliminary analyses. No main effects for gender or course of study could be found for goals, emotions or achievement. Therefore, gender and course of study were not included in the main analyses, and results are presented for the whole sample.

### Construct Validity of the 3 × 2 Achievement Goal Model

**Table 1** provides descriptive statistics, internal consistencies and composite reliability of the achievement goal variables. Our findings showed that the internal consistencies and values of composite reliability for all scales ranged from moderate to excellent (0.64–0.94). **Table 2** provides intercorrelations among the achievement goal variables.

**Table 1:** Psychometric properties of achievement goals, emotions and performance.

				Range				
Scale	*k*	*M*	*SD*	Potential	Actual	α	CR	95% CI CR
Task-approach goals	3	6.59	0.74	1–7	2.0–7.0	0.84	0.84	0.77–0.91
Task-avoidance goals	3	6.19	1.06	1–7	2.0–7.0	0.64	0.65	0.56–0.74
Self-approach goals	3	3.89	1.75	1–7	1.0–7.0	0.88	0.88	0.85–0.91
Self-avoidance goals	3	4.27	1.74	1–7	1.0–7.0	0.84	0.84	0.80–0.87
Other-approach goals	3	2.95	1.73	1–7	1.0–7.0	0.94	0.94	0.93–0.96
Other-avoidance goals	3	2.89	1.70	1–7	1.0–7.0	0.91	0.91	0.89–0.93
Enjoyment	8	2.55	0.77	1–5	1.0–4.9	0.88	0.89	0.87–0.90
Boredom	8	2.36	0.93	1–5	1.0–5.0	0.93	0.93	0.92–0.95
Academic achievement	–	3.03	0.97	1–5	1.0–5.0	–	–	–

*N = 388; k = number of items; CR, composite reliability (Raykov, 2009).*

**Table 2:** Intercorrelations among the achievement goal variables, emotions, and achievement.

	1	2	3	4	5	6	7	8	9
(1) Task-approach goals	–								
(2) Task-avoidance goals	0.54	–							
(3) Self-approach goals	0.14	0.12	–						
(4) Self-avoidance goals	0.12	0.24	0.67	–					
(5) Other-approach goals	–0.05	–0.09	0.30	0.20	–				
(6) Other-avoidance goals	–0.10	–0.02	0.30	0.25	0.85	–			
(7) Enjoyment	0.17	0.04	0.32	0.16	0.25	0.17	–		
(8) Boredom	–0.17	–0.03	–0.08	–0.03	0.01	0.06	–0.52	–	
(9) Achievement	0.17	0.05	0.06	0.12	0.16	0.07	0.13	0.04	–

*N = 388; | r|≥ 0.10, p < 0.05.*

A CFA was conducted to examine the construct validity of the 3 × 2 goal model. The results of the CFAs provided strong support for the proposed structure of the 3 × 2 goal model. Model fit indices showed a good model fit, χ^2^(120, *N* = 388) = 336.40, *p* < 0.01, CFI = 0.949, TLI = 0.935, RMSEA = 0.057, SRMR = 0.037. All standardized factor loadings were moderate to strong (ranging from 0.44 to 0.93).

Additionally, we constructed five alternative models (unidimensional goal model, dichotomous, trichotomous, 2 × 2) and tested them competitively, aiming to show the distinctness of the different achievement goal conceptualizations. Model comparison with descriptive measures of model parsimony (AIC, BIC) showed a better fit for the 3 × 2 model, AIC = 21563.62, BIC = 21836.93, than any of the alternative models (see **Table 3**). Model estimation and model comparison revealed inacceptable fit indices for the five alternative models.

**Table 3:** Comparison of the achievement goal conceptualizations.

Model	χ^2^ (*N* = 388)	df	CFI	TLI	RMSEA	90% CI RMSEA	SRMR	AIC	BIC
3 × 2 model	268.99∗	120	0.949	0.935	0.057	0.048–0.066	0.037	21563.62	21836.93
2 × 2 model	1602.06∗	129	0.497	0.404	0.172	0.164–0.179	0.180	22836.63	23074.29
Trichotomous model	874.85∗	132	0.746	0.706	0.120	0.113–0.128	0.137	22512.01	22737.79
Dichotomous model	1474.83∗	134	0.542	0.477	0.161	0.153–0.168	0.183	23011.46	23229.31
Unidimensional goal model	1614.99∗	135	0.495	0.427	0.168	0.161–0.175	0.204	23723.04	23936.93
Definition model	467.06∗	132	0.886	0.867	0.081	0.073–0.089	0.052	21818.82	22044.60

*CFI, comparative fit index; TLI, Tucker-Lewis index; RMSEA, root mean square error of approximation; SRMR, standardized root mean square residual; AIC, Akaike information criterion; BIC, Bayesian information criterion; MLR, maximum likelihood robust estimation in Mplus was used in all analyses; ∗p < 0.001.*

### Joint and Mediated Effects of Goals and Emotion on Achievement

Multiple mediator modeling with latent variables was employed to investigate the associations between goals, emotions and academic achievement. Preliminary analyses were conducted to control for possible multicollinearities between the six goals that were used as predictors. The variance inflation factor for the achievement goal variables ranged from 1.49 to 3.86 (below the conventional cutoff criteria of 10; Kutner et al., 2004) indicating that multicollinearity for the six goals as predictors of enjoyment and boredom was not high but the regression may be still biased (Bowerman and O’Connell, 1990). Additionally, the correlations between the latent goal variable pairs of other (0.93), self-goals (0.82), task goals (0.78) were very high. To avoid untrustworthy estimates and standard errors due to multicollinearities we conducted two separate models: one including all three approach goals and one including all three avoidance goals.

#### Approach Goal Multiple Mediator Model

As can be seen in **Table 4**, the multiple mediator model with direct associations between approach goals and student achievement fits the data well and showed better model fit than the model without direct associations between approach goals and achievement. The results of this model are displayed in **Table 5**. Task-approach (*b∗* = 0.153, *SE* = 0.055, *p* = 0.006), self-approach (*b*∗ = −0.275, *SE* = 0.061, *p* < 0.001) and other–approach (*b*∗ = 0.164, *SE* = 0.057, *p* = 0.004) were shown to predict enjoyment. Only task-approach negatively predicted boredom (b* = −0.176, *SE* = 0.072, *p* = 0.014). Task-approach (*b*∗ = 0.178, *SE* = 0.060, *p* = 0.003) and other-approach (*b*∗ = 0.168, *SE* = 0.060, *p* = 0.005) directly predicted achievement. No emotion construct predicted achievement. This was the only result that differed between the two multiple mediator models: through including direct associations between goals and achievement, the formerly moderate positive link between enjoyment and achievement (*b*∗ = 0.166; *SE* = 0.078; *p* = 0.034) was no longer significant.

**Table 4:** Comparison of the approach and avoidance multiple mediator models.

Model	χ^2^ (*N* = 388)	df	CFI	TLI	RMSEA	90% CI RMSEA	SRMR	AIC	SABIC
**Approach goals**									
Multiple mediator model (without direct association)	563.80∗	288	0.946	0.939	0.050	0.044–0.056	0.044	26317.90	26388.04
Multiple mediator model (with direct association)	548.75∗	285	0.948	0.941	0.049	0.043–0.055	0.041	26307.38	26379.88
**Avoidance goals**									
Multiple mediator model (without direct association)	579.41∗	288	0.939	0.931	0.051	0.045–0.057	0.047	28289.87	28360.01
Multiple mediator model (with direct association)	572.83∗	285	0.939	0.931	0.051	0.045–0.057	0.046	28289.08	28361.59

*CFI, comparative fit index; TLI, Tucker-Lewis index; RMSEA, root mean square error of approximation; SRMR, standardized root mean square residual; AIC, Akaike information criterion; SABIC, sample-size adjusted Bayesian information criterion; MLR, maximum likelihood robust estimation in Mplus was used in all analyses; ∗p < 0.001.*

**Table 5:** Multiple mediator models.

	Mediators			Outcome
Predictor variables	Enjoyment	Boredom		Achievement
**Model approach goals (with direct associations)**		
Task-approach	0.153 (0.055)**	–0.176 (0.072)*		0.178 (0.060)**
Self-approach	0.275 (0.061)***	–0.078 (0.063)		–0.034 (0.061)
Other-approach	0.164 (0.057)**	0.030 (0.056)		0.168 (0.060)**
Enjoyment	–	–		0.084 (0.085)
Boredom	–	–		0.031 (0.078)
**Model avoidance goals (without direct associations)**				
Task-avoidance	–0.026 (0.091)	–0.048 (0.070)		–
Self-avoidance	0.149 (0.071)*	–0.037 (0.085)		–
Other-avoidance	0.117 (0.059)*	0.079 (0.058)		–
Enjoyment	–	–		0.160 (0.078)∗
Boredom	–	–		0.050 (0.075)

∗*p ≤ 0.05, ∗∗p ≤ 0.01, ∗∗∗p ≤ 0.001, values are standardized parameter estimates, values in parentheses are standard errors (SE).*

#### Avoidance Goal Multiple Mediator Model

Both avoidance goal multiple mediator models fit the data well and showed almost identical model fit statistics (see **Table 4**). Therefore, we additionally conducted the χ^2^ difference tests with the Satorra-Bentler scaling correction for non-normal data. As the result showed no significant differences between the two models [χ^2^(3) = 6.585, *p* = 0.086] we followed conventional guidelines (*lex parsimoniae*) and selected the more parsimonious model without direct associations (see **Table 5**). In this model self-avoidance (*b*∗ = 0.149, *SE* = 0.071, *p* = 0.036) and other-avoidance (*b*∗ = 0.117, *SE* = 0.059, *p* = 0.047) were shown to predict enjoyment. No goal type predicted boredom. Only enjoyment predicted achievement (*b*∗ = 0.160, *SE* = 0.078, *p* = 0.039). This was the only result that differed between the two avoidance multiple mediator models: when considering also direct associations between goals and achievement, the moderate positive link between enjoyment and achievement is no longer significant. In the model with direct associations no avoidance goal type were shown to predict academic achievement.

#### Indirect Effects

The indirect effects of achievement goals on achievement by way of emotion variables were assessed using bias-corrected bootstrapping (MacKinnon et al., 2004), with estimates of 1000 bootstrap samples (95% confidence intervals). No mediation effects were found (see **Table 6**).

**Table 6:** Tests of significance of mediation.

			Original sample		Bootstrap	
Independent variable	Mediating variable	Dependent variable	Standardized indirect effect	*SE*	Mean indirect effect^b^	95% CI with bias correction (upper, lower)
Task-approach	Emotions^a^	Achievement	0.007	0.012	0.011	–0.026, 0.054
Self-approach	Emotions^a^	Achievement	0.021	0.021	0.011	–0.013, 0.035
Other-approach	Emotions^a^	Achievement	0.015	0.016	0.009	–0.011, 0.026
						
Task-avoidance	Emotions^a^	Achievement	–0.005	0.010	–0.006	–0.041, 0.014
Self-avoidance	Emotions^a^	Achievement	0.017	0.013	0.011	–0.004, 0.029
Other-avoidance	Emotions^a^	Achievement	0.016	0.016	0.011	–0.009, 0.032

^a^*Mplus bootstrap confidence intervals refer to the significance of the total indirect effect of initial goals on achievement as mediated by both boredom and enjoyment.*^b^*These values are based on unstandardized mean path coefficients.*

## Discussion

The main aims of the present study were to test the recently proposed 3 × 2 model of achievement goals and to investigate its associations with activity emotions (enjoyment, boredom) and academic achievement in a comprehensive model. Replicability is an important and indispensable principle in psychology (Asendorpf et al., 2013) as well as in educational research in general (Schneider, 2004). Replicability is necessary to provide valid scientific results that can be generalized to more people and settings than are represented in existing studies. We were able to replicate the finding of previous research (Elliot et al., 2011; Diseth, 2015) regarding the construct validity of the 3 × 2 model with a similar sample in a different context (scientific methods). We found no gender differences for goals in our preliminary analyses. Even if some authors found gender differences in previous studies using the 2 × 2 model (e.g., Meece et al., 2006 with school children), Elliot and McGregor (2001) only found them for mastery approach goals in one of their studies. However, in recent studies using the 3 × 2 model, either gender effects have not been addressed (e.g., Johnson and Kestler, 2013; Diseth, 2015), or no gender effects have been found (e.g., Mascret et al., 2015a), which is in line with our finding. Nevertheless, it would be an interesting issue for further research to focus on the question of gender effects in the 3 × 2 model.

We also investigated the associations between the new achievement goal types, achievement emotions and academic achievement. This is crucial, as previous research (e.g., Pekrun et al., 2009, 2014a; Huang, 2011) has shown complex links between students’ goals (using a trichotomous or 2 × 2 goal conceptualization) and emotional experience. The general pattern of positive academic emotions corresponding to approach goals could be also found in our sample: task-approach, self-approach and other approach goals are positively related to enjoyment. The finding that other-approach goals are also beneficial for students’ emotional experience while learning for exams is not well established so far. However, this result is in concordance with prior research (Pekrun et al., 2014b) and it can be potentially explained with the exam context in this study where performance contingencies were particularly salient. Furthermore, only task-approach goals are negatively related to boredom which is in line with previous results on the link of mastery approach goals and boredom (Daniels et al., 2009). The general pattern of negative emotions corresponding to avoidance goals could not be found in our sample. Contrary to previous empirical results (e.g., Pekrun et al., 2009; Huang, 2011), no avoidance goal type predicted boredom. Additionally, the finding that other-avoidance goals are positively related to enjoyment is surprising as former studies found no association at all for these variables (Pekrun et al., 2006, 2009). A new result is the positive link of self-avoidance and enjoyment. However, these associations between avoidance goals and enjoyment are only moderate and lower than the associations between approach goals and enjoyment. There is a clear need for additional research on avoidance goals and emotions.

After controlling for approach and avoidance goals, direct associations between goals and achievement could be only found for task-approach and other-approach goals, both of which had positive associations with exam performance. The finding for other-approach goals is in line with previous research (Elliot et al., 2011; Diseth, 2015), whereas the association between task-approach goals and achievement is not well established (Elliot et al., 2011), and additional research is needed to further investigate and validate this finding. The lack of association between other-avoidance goals and achievement was also found in one recent empirical study (Diseth, 2015). Confirming previous research, enjoyment is positively related to achievement. However, in our study boredom is not linked to achievement, a result which is not in line with a recent meta-analysis showing an overall negative correlation of −0.25 (Tze et al., 2016). After controlling for goals and direct associations between goals and achievement, both emotions were not predictors of exam performance.

To answer the third research question, we considered criticisms of the classical mediation framework (Baron and Kenny, 1986) and used structural equation modeling and bootstrapping to investigate mediational effects. Indirect effects for the activity emotions of enjoyment and boredom could not be found with regard to other-based goals. This is in line with previous findings based on a classical mediation approach (Pekrun et al., 2009).

### Limitations and Future Research

Three limitations of this study should be noted. First, descriptive statistics (mean, standard deviation, and range) suggest that there is a *ceiling effect* for task-approach and task-avoidance goals in this sample. The mean is very high and there is not much variance to be explained in comparison to the other goal types. Second, the bivariate correlation between the pairs of task-based, self-based, and other-based goals are high indicating a problem of multicollinearity. Theoretically the high correlation between goal pairs are to be expected because (1) they each share a competence-based component and (2) are commonly measured with items containing substantial semantic overlap (as the AGQ in this study). Empirical evidence, however, provides good reasons to keep them separated (Murayama et al., 2011). To solve this issue of multicollinearity we conducted two separate models for approach goals and avoidance goals. Thus, we were able to keep the focus on a greater number of goals as conceptualized in the 3 × 2 model. Unfortunately, with our procedure we were not able to consider the relationships between all six goals in one comprehensive model. As there is no simple answer how to deal in a traditional variable-centered approach with the problem of multicollinearity between goal pairs we propose that further research should also include person-centered approaches focusing on a multiple goal perspective (Linnenbrink-Garcia and Barger, 2014). Third, our self-report measures targeted students’ experiences in their scientific methods lecture at one university. The peculiarities of the sample selected restrict the generalizability of the results.

Future research will need to test the 3 × 2 model in other samples of students varying by lecture content, age (e.g., elementary, middle, high school, college, and university students), other standardized achievement outcomes, and culture (e.g., in an Asian context). Additionally, future studies should also investigate the relationship between the 3 × 2 model and other achievement emotions such as hope, pride, relief, anger, anxiety, shame, and hopelessness.

### Practical Implications

Finally, the findings suggest the merits of approach goals compared to avoidance goals in terms of facilitating students’ enjoyment. The beneficial effects of self-approach goals and task-approach goals on students’ emotional experience while learning for exams represent one more argument in favor of focusing on goals that relate on improving students’ level of competence as a crucial determinant of their development of lifelong learning competencies (Lüftenegger et al., 2012; Schober et al., 2013).

## Author Contributions

All listed authors contributed meaningfully to the paper. ML, KH, and MLa developed the study concept. All authors contributed to the study design, analyzed or interpreted the data. ML, KH, and MLa prepared the draft manuscript, and JK, CS, and BS provided critical revisions. All authors approved the final version of the manuscript for submission.

## Conflict of Interest Statement

The authors declare that the research was conducted in the absence of any commercial or financial relationships that could be construed as a potential conflict of interest.

## References

[B1] Ainley M. Hidi S. (2014). “Interest and enjoyment,” in *International Handbook of Emotions in Education*, eds Pekrun R. Linnenbrink-Garcia L. (New York, NY: Routhledge), 205–227.

[B2] American Psychological Association [APA]. (2010). *Publication Manual of the American Psychological Association*, 6th Edn. Washington, DC: American Psychological Association.

[B3] Ames C. (1992). Classrooms: goals, structures, and student motivation. *J. Educ. Psychol.* 84 261–271. 10.1037/0022-0663.84.3.261

[B4] Anderman E. M. Wolters C. A. (2006). “Goals, values, and affect: influences on student motivation,” in *Handbook of Educational Psychology*, eds Alexander P. A. Winne P. H. (Mahwah, NJ: Lawrence Erlbaum Associates Publishers), 369–389.

[B5] Asendorpf J. B. Conner M. De Fruyt F. De Houwer J. Denissen J. J. A. Fiedler K. (2013). Recommendations for increasing replicability in psychology. *Eur. J. Pers.* 27 108–119. 10.1002/per.1919

[B6] Baron R. M. Kenny D. A. (1986). The moderator–mediator variable distinction in social psychological research: conceptual, strategic, and statistical considerations. *J. Pers. Soc. Psychol.* 51:1173 10.1037/0022-3514.51.6.1173 3806354

[B7] Bowerman B. L. O’Connell R. T. (1990). *Linear Statistical Models: An Applied Approach*, 2nd Edn. Belmont, CA: Duxbury.

[B8] Bundesministerium für Gesundheit. (1995). *Ethikrichtlinie für Klinische Psychologinnen und Klinische Psychologen sowie für Gesundheitspsychologinnen und Gesundheitspsychologen [Ethical Guidelines for Clinical and Health Psychologists]*. Wien: Bundesministerium für Gesundheit.

[B9] Burnham K. P. Anderson D. R. (2004). Muldimodel inference: understanding AIC and BIC in model selection. *Sociol. Methods Res.* 33 261–304. 10.1177/0049124104268644

[B10] Daniels L. M. Haynes T. L. Stupnisky R. H. Perry R. P. Newall N. E. Pekrun R. (2008). Individual differences in achievement goals: a longitudinal study of cognitive, emotional, and achievement outcomes. *Contemp. Educ. Psychol.* 33 584–608. 10.1016/j.cedpsych.2007.08.002

[B11] Daniels L. M. Stupnisky R. H. Pekrun R. Haynes T. L. Perry R. P. Newall N. E. (2009). A longitudinal analysis of achievement goals: from affective antecedents to emotional effects and achievement outcomes. *J. Educ. Psychol.* 101 948–963. 10.1037/a0016096

[B12] Diseth, Å (2015). The advantages of task-based and other-based achievement goals as standards of competence. *Int. J. Educ. Res.* 72 59–69. 10.1016/j.ijer.2015.04.011

[B13] Dweck C. S. (1986). Motivational process affects learning. *Am. Psychol.* 41 1040–1048. 10.1037/0003-066X.41.10.1040

[B14] Dweck C. S. Leggett E. L. (1988). A social-cognitive approach to motivation and personality. *Psychol. Rev.* 95 256–273. 10.1037/0033-295X.95.2.256

[B15] Elliot A. J. (1999). Approach and avoidance motivation and achievement goals. *Educ. Psychol.* 34 169–189. 10.1207/s15326985ep3403_3

[B16] Elliot A. J. (2005). “Goals a conceptual history of the achievement goal construct,” in *Handbook of Compentence and Motivation*, eds Elliot A. J. Dweck C. S. (New York, NY: The Guilford Press), 52–72.

[B17] Elliot A. J. Harackiewicz J. M. (1996). Approach and avoidance achievement goals and intrinsic motivation: a mediational analysis. *J. Pers. Soc. Psychol.* 70 461–475. 10.1037/0022-3514.70.3.461 8014838

[B18] Elliot A. J. McGregor H. A. (1999). Test anxiety and the hierarchical model of approach and avoidance achievement motivation. *J. Pers. Soc. Psychol.* 76 628–644. 10.1037/0022-3514.76.4.628 10234849

[B19] Elliot A. J. McGregor H. A. (2001). A 2 x 2 achievement goal framework. *J. Pers. Soc. Psychol.* 80 501–519. 10.1037//0022-3514.80.3.501 11300582

[B20] Elliot A. J. Murayama K. Pekrun R. (2011). A 3 × 2 achievement goal model. *J. Educ. Psychol.* 103 632–648. 10.1037/a0023952

[B21] Elliot A. J. Thrash T. M. (2002). Approach-avoidance motivation in personality: approach and avoidance temperaments and goals. *J. Pers. Soc. Psychol.* 82 804–818. 10.1037//0022-3514.82.5.804 12003479

[B22] Gillet N. Lafrenière M.-A. K. Huyghebaert T. Fouquereau E. (2015). Autonomous and controlled reasons underlying achievement goals: Implications for the 3 × 2 achievement goal model in educational and work settings. *Motivat. Emot.* 39 858–875. 10.1007/s11031-015-9505-y

[B23] Goetz T. Hall N. C. (2013). “Emotion and achievement in the classroom,” in *International Guide to Student Achievement*, eds Hattie J. A. C. Anderman E. M. (New York, NY: Routledge), 192–195.

[B24] Goetz T. Sticca F. Pekrun R. Murayama K. Elliot A. J. (2016). Intraindividual relations between achievement goals and discrete achievement emotions: an experience sampling approach. *Learn. Instruct.* 41 115–125. 10.1016/j.learninstruc.2015.10.007

[B25] Huang C. (2011). Achievement goals and achievement emotions: a meta-analysis. *Educ. Psychol. Rev.* 23 359–388. 10.1007/s10648-011-9155-x

[B26] Hulleman C. Schrager S. M. Bodmann S. M. Harackiewicz J. M. (2010). A meta-analytic review of achievement goal measures: different labels for the same constructs or different constructs with similar labels? *Psychol. Bull.* 136 422–449. 10.1037/a0018947 20438145

[B27] Johnson M. L. Kestler J. L. (2013). Achievement goals of traditional and nontraditional aged college students: using the 3 × 2 achievement goal framework. *Int. J. Educ. Res.* 61 48–59. 10.1016/j.ijer.2013.03.010

[B28] King R. B. McInerney D. M. Watkins D. A. (2012). How you think about your intelligence determines how you feel in school: the role of theories of intelligence on academic emotions. *Learn. Individ. Differ.* 22 814–819. 10.1016/j.lindif.2012.04.005

[B29] Kline R. B. (2011). *Principles and Practice of Structural Equation Modeling*, 3rd Edn. New York, NY: Guilford Press.

[B30] Krapp A. (2002). Structural and dynamic aspects of interest development: theoretical considerations from an ontogenetic perspective. *Learn. Instruct.* 12 383–409. 10.1016/S0959-4752(01)00011-1

[B31] Kutner M. H. Nachtsheim C. J. Neter J. (2004). *Applied Linear Regression Models.* Boston, MA: McGraw-Hill.

[B32] Lance C. E. Butts M. M. Michels L. C. (2006). The sources of four commonly reported cutoff criteria what did they really say? *Organ. Res. Methods* 9 202–220. 10.1177/1094428105284919

[B33] Linnenbrink E. A. Pintrich P. R. (2002). Achievement goal theory and affect: an asymmetrical bidirectional model. *Educ. Psychol.* 37 69–78. 10.1207/S15326985EP3702_2

[B34] Linnenbrink E. A. Ryan A. M. Pintrich P. R. (1999). The role of goals and affect in working memory functioning. *Learn. Individ. Differ.* 11 213–230. 10.1016/S1041-6080(00)80006-0

[B35] Linnenbrink-Garcia L. Barger M. M. (2014). “Achievement goals and emotions,” in *International Handbook of Emotions in Education*, eds Pekrun R. Linnenbrink-Garcia L. (New York, NY: Routledge), 142–161.

[B36] Linnenbrink-Garcia L. Tyson D. Patall E. (2008). When are achievement goal orientations beneficial for academic achievement? A closer look at main effects and moderating factors. *Rev. Int. Psychol. Soc.* 21 19–70.

[B37] Lüftenegger M. Schober B. van de Schoot R. Wagner P. Finsterwald M. Spiel C. (2012). Lifelong learning as a goal - do autonomy and self-regulation in school result in well prepared pupils? *Learn. Instruct.* 22 27–36. 10.1016/j.learninstruc.2011.06.001

[B38] MacKinnon D. P. Lockwood C. M. Hoffman J. M. West S. G. Sheets V. (2002). A comparison of methods to test mediation and other intervening variable effects. *Psychol. Methods* 7 83–104. 10.1037//1082-989X.7.1.83 11928892 PMC2819363

[B39] MacKinnon D. P. Lockwood C. M. Williams J. (2004). Confidence limits for the indirect effect: distribution of the product and resampling methods. *Multiv. Behav. Res.* 39 99–128. 10.1207/s15327906mbr3901_4 PMC2821115 20157642

[B40] Maehr M. L. (1989). “Thoughts about motivation,” in *Research on Motivation in Education* Vol. 3 eds Ames C. Ames R. (New York, NY: Academic Press), 299–315.

[B41] Martin A. J. (2013). “Goal orientation,” in *International Guide to Student Archievement*, eds Hattie J. Anderman E. M. (New York, NY: Routledge), 353–355.

[B42] Mascret N. Elliot A. J. Cury F. (2015a). Extending the 3 × 2 achievement goal model to the sport domain: the 3 × 2 achievement goal questionnaire for sport. *Psychol. Sport Exercise* 17 7–14. 10.1016/j.psychsport.2014.11.001

[B43] Mascret N. Elliot A. J. Cury F. (2015b). The 3 × 2 achievement goal questionnaire for teachers. *Educ. Psychol.* 10.1080/01443410.2015.1096324

[B44] Meece J. L. Glienke B. B. Burg S. (2006). Gender and motivation. *J. Sch. Psychol.* 44 351–373. 10.1016/j.jsp.2006.04.004

[B45] Murayama K. Elliot A. J. Yamagata S. (2011). Separation of performance-approach and performance-avoidance achievement goals: a broader analysis. *J. Educ. Psychol.* 103 238–256. 10.1037/a0021948

[B46] 1998–2015 Muthén B. O. Muthén L. K. (1998–2015). *Mplus (Version 7.33)*. Los Angeles, CA: Muthén & Muthén.

[B47] Nicholls J. G. (1984). Achievement motivation: conceptions of ability, subjective experience, task choice, and performance. *Psychol. Rev.* 91 328–346. 10.1037/0033-295X.91.3.328

[B48] Open Science Collaboration (2015). Estimating the reproducibility of psychological science. *Science* 349:aac4716 10.1126/science.aac4716 26315443

[B49] Pekrun R. (2006). The control-value theory of achievement emotions: assumptions, corollaries, and implications for educational research and practice. *Educ. Psychol. Rev.* 18 315–341. 10.1007/s10648-006-9029-9

[B50] Pekrun R. Cusack A. Murayama K. Elliot A. J. Thomas K. (2014a). The power of anticipated feedback: effects on students’ achievement goals and achievement emotions. *Learn. Instruct.* 29 115–124. 10.1016/j.learninstruc.2013.09.002

[B51] Pekrun R. Elliot A. J. Maier M. A. (2006). Achievement goals and discrete achievement emotions: a theoretical model and prospective test. *J. Educ. Psychol.* 98 583–597. 10.1037/0022-0663.98.3.583

[B52] Pekrun R. Elliot A. J. Maier M. A. (2009). Achievement goals and achievement emotions: testing a model of their joint relations with academic performance. *J. Educ. Psychol.* 101 115–135. 10.1037/a0013383

[B53] Pekrun R. Goetz T. Frenzel A. C. Barchfeld P. Perry R. P. (2011). Measuring emotions in students’ learning and performance: the achievement emotions questionnaire (AEQ). *Contemp. Educ. Psychol.* 36 36–48. 10.1016/j.cedpsych.2010.10.002

[B54] Pekrun R. Goetz T. Titz W. Perry R. P. (2002). Academic emotions in students’ self-regulated learning and achievement: a program of qualitative and quantitative research. *Educ. Psychol.* 37 91–105. 10.1207/S15326985EP3702_4

[B55] Pekrun R. Hall N. C. Goetz T. Perry R. P. (2014b). Boredom and academic achievement: testing a model of reciprocal causation. *J. Educ. Psychol.* 106 696–710. 10.1037/a0036006

[B56] Preacher K. J. Hayes A. F. (2008). Asymptotic and resampling strategies for assessing and comparing indirect effects in multiple mediator models. *Behav. Res. Methods* 40 879–891. 10.3758/BRM.40.3.879 18697684

[B57] Putwain D. W. Larkin D. Sander P. (2013). A reciprocal model of achievement goals and learning related emotions in the first year of undergraduate study. *Contemp. Educ. Psychol.* 38 361–374. 10.1016/j.cedpsych.2013.07.003

[B58] Raykov T. (2009). Evaluation of scale reliability for unidimensional measures using latent variable modeling. *Measur. Evalu. Counsel. Dev.* 42 223–232. 10.1177/0748175609344096

[B59] Schafer J. L. Graham J. W. (2002). Missing data: our view of the state of the art. *Psychol. Methods* 7 147–177. 10.1037/1082-989X.7.2.147 12090408

[B60] Schiefele U. Winteler A. Krapp A. (1988). Studieninteresse und fachbezogene Wissensstruktur (Study interest and the structure of subject matter related knowledge. *Psychol. Erzieh. Unterricht* 35 106–118.

[B61] Schneider B. (2004). Building a scientific community: the need for replication. *Teach. Col. Record* 106 1471–1483. 10.1111/j.1467-9620.2004.00386.x

[B62] Schober B. Lüftenegger M. Wagner P. Finsterwald M. Spiel C. (2013). Facilitating lifelong learning in school-age learners: programs and recommendations. *Eur. Psychol.* 18 114–125. 10.1027/1016-9040/a000129

[B63] Schunk D. H. Pintrich P. R. Meece J. L. (2008). *Motivation in Education: Theory, Research, and Applications*. Upper Saddle River, NJ: Pearson/Merrill Prentice Hall.

[B64] Schwarz G. (1978). Estimating the dimension of a model. *Ann. Stat.* 6 461–465. 10.1214/aos/1176344136

[B65] Seifert T. L. (1995). Academic goals and emotions: a test of two models. *J. Psychol.* 129:543 10.1080/00223980.1995.9914926

[B66] Stoeber J. Haskew A. E. Scott C. (2015). Perfectionism and exam performance: the mediating effect of task-approach goals. *Pers. Individ. Differ.* 74 171–176. 10.1016/j.paid.2014.10.016

[B67] Thorkildsen T. A. Nicholls J. G. (1998). Fifth Graders’ achievement orientations and beliefs: individual and classroom differences. *J. Educ. Psychol.* 90 179–201. 10.1037/0022-0663.90.2.179

[B68] Tze V. M. C. Daniels L. M. Klassen R. M. (2016). Evaluating the relationship between boredom and academic outcomes: a meta-analysis. *Educ. Psychol. Rev.* 28 119–144. 10.1007/s10648-015-9301-y

[B69] Zhao X. Lynch J. G. Jr. Chen Q. (2010). Reconsidering baron and kenny: myths and truths about mediation analysis. *J. Consumer Res.* 37 197–206. 10.1086/651203

